# Development of a Topical Treatment for Psoriasis Targeting RORγ: From Bench to Skin

**DOI:** 10.1371/journal.pone.0147979

**Published:** 2016-02-12

**Authors:** Susan H. Smith, Carlos E. Peredo, Yukimasa Takeda, Thi Bui, Jessica Neil, David Rickard, Elizabeth Millerman, Jean-Philippe Therrien, Edwige Nicodeme, Jean-Marie Brusq, Veronique Birault, Fabrice Viviani, Hans Hofland, Anton M. Jetten, Javier Cote-Sierra

**Affiliations:** 1 Discovery and Preclinical Development, Stiefel, a GSK company, Research Triangle Park, North Carolina, United States of America; 2 Immunity, Inflammation and Disease Laboratory, National Institute of Environmental Health Sciences, National Institutes of Health, Research Triangle Park, North Carolina, United States of America; 3 Flexible Discovery Unit, GlaxoSmithKline, Les Ulis Cedex, France; 4 MDR/PTS, GlaxoSmithKline, Research Triangle Park, North Carolina, United States of America; 5 Respiratory Therapeutic Area, GlaxoSmithKline, Medicines Research Centre, Gunnels Wood Road, Stevenage, Hertfordshire, United Kingdom; University of Alabama at Birmingham, UNITED STATES

## Abstract

**Background:**

Psoriasis is a chronic inflammatory skin disorder involving marked immunological changes. IL-17-targeting biologics have been successful in reducing the disease burden of psoriasis patients with moderate-to-severe disease. Unfortunately, the stratum corneum prevents penetration of large molecule weight proteins, including monoclonal antibodies. Thus, for the majority of psoriasis patients ineligible for systemic treatments, a small molecule targeting RORγt, the master regulator of IL-17 family cytokines, may represent an alternative topical medicine with biologic-like efficacy.

**Methods and Findings:**

The preclinical studies described in this manuscript bridge the gap from bench to bedside to provide the scientific foundation for a compound entering clinical trials for patients with mild to moderate psoriasis. In addition to several ex vivo reporter assays, primary T cell cultures, and the imiquimod mouse model, we demonstrate efficacy in a newly developed human ex vivo skin assay, where Th17-skewed cytokine expression is induced from skin-resident immune cells. Importantly, the skin barrier remains intact allowing for the demonstration of topical drug delivery. With the development of this novel assay, we demonstrate potent compound activity in the target tissue: human skin. Finally, target engagement by this small molecule was confirmed in *ex vivo* lesional psoriatic skin.

**Conclusions:**

Our work describes a progressive series of assays to demonstrate the potential clinical value of a novel RORγ inverse agonist small molecule with high potency and selectivity, which will enter clinical trials in late 2015 for psoriasis patients.

## Introduction

There has been much progress in our understanding of psoriasis immunopathology, which has contributed to the development of new and effective biologic and systemic drugs patients. Psoriasis vulgaris is a chronic autoimmune inflammatory skin disorder that results from a complex interaction of genetic, environmental and systemic factors and affects 2–3% of the Caucasian population [1]. Immune system dysregulation is implicated in disease pathogenesis; inflammatory cell infiltrates in psoriatic lesions consist of innate and adaptive immune cells and the inflammatory cytokines and chemokines produced by infiltrating leukocytes drive the epidermal changes characteristic of psoriatic plaques. For instance, Th17-type cytokines (IL-17A, IL-17F and IL-22) drive keratinocyte hyperproliferation and chemokine production, and perpetuate further leukocyte recruitment [2,3]. The central importance of IL-17 to the development and maintenance of disease has been confirmed with the clinical effectiveness of IL-17/IL-17 receptor neutralizing antibodies in psoriasis patients [4,5], where systemic treatment with these biologics normalizes inflammatory gene expression [6,7]. Unfortunately, the large molecular weight of antibodies makes them unsuitable for development as topical medicines because they cannot diffuse across the skin barrier. Thus, despite many advances, few if any effective novel topical medicines have been developed for the vast majority of psoriasis patients with mild-to-moderate disease, who may not be candidates for systemic or biologic therapy. Toward this aim, we have developed and are progressing to human clinical trials a novel topical RORγ inverse agonist that has the potential to provide to patients a topical medicine with a mechanism of action that suggests it may yield the efficacy of an IL-17 biologic.

Within psoriatic lesions, IL-17 can be produced by several cell type, including Th17 cells (the most extensively studied), γδ T cells, innate lymphoid cells (ILCs), a subpopulation of activated epidermal CD8+ T cells, neutrophils and possibly mast cells [8–11]. Although several transcription factors may be important, the development and maintenance of IL-17 producing cells is controlled by a master regulator transcription factor, the nuclear receptor retinoid-related orphan receptor (RORγt) [12–14]. RORγt is both necessary and sufficient for IL-17 transcription and Th17 lineage differentiation in both human and mice [12,13,15] and T cells from RORγt knockout mice are greatly attenuated in their *in vitro* differentiation into Th17 cells [16].

While RORγt expression is largely restricted to hematopoietic cell lineages, the long isoform, RORγ, is widely expressed and plays important roles in development, inflammation, lipid and glucose metabolism and circadian rhythm [17]. Several synthetic ligands have been developed to probe RORγ/RORγt as a *bona fide* drug target for the treatment of several human diseases, including autoimmune diseases, metabolic disorders, behavioral and sleep disorders, and IL-17-driven inflammatory diseases [18,19]. RORγt differs from RORγ in the first 100 nucleotides, but share the same DNA and ligand binding domains; thus, systemic treatments aimed at treating inflammation with RORγt inhibition may incur unwanted side effects through cross-reactivity with RORγ. In psoriasis, as with other inflammatory skin disorders, the target tissue is readily accessible. Therefore, local inhibition of RORγ/RORγt with small molecular weight compounds represents a unique opportunity to selectively inhibit aberrant IL-17 cytokine production in the plaque while limiting systemic exposure.

In this report, we describe a novel, potent and highly selective small molecule inhibitor for RORγ/RORγt, that markedly inhibits Th17-type cytokine production in multiple assay systems, including (i) *in vitro* reporter assays, (ii) the *in vivo* imiquimod mouse model, and (iii) human tissue-based assays, including human peripheral T cells, Th17-skewed *ex vivo* human skin and psoriatic biopsy cultures from psoriasis patients. Based on these supporting data, we are progressing this RORγ-specific inverse agonist to clinical trials for topical treatment of mild to moderate psoriasis, expecting that it will impact local cytokine expression and lead to a positive clinical response for patients.

## Materials and Methods

### Tissue Acquisition

All human biological samples were sourced ethically and their research use was in accord with the terms of the informed consents. For full thickness human skin, the acquisition, informed consent form (IFC), and protocol for use were approved by an independent Investigational Review Board (Pearl IRB, Indianapolis, IN).

All animal studies were ethically reviewed and carried out in accordance with European Directive 86/609/EEC and the GSK Policy on the Care, Welfare and Treatment of Animals. The protocol was approved by the Committee on the Ethics of Animal Experiments of the GSK France Research Centre, registered as CEEA-25 (Protocol Permit Number: 2012–10). Animal welfare was recorded every day and all efforts were made to minimize suffering. At the end of the study, euthanasia was performed by exsanguination and cervical dislocation under 3–5% isoflurane anesthesia.

### Reporter gene assay

Doxycycline-inducible ROR stable cell lines were generated by transfecting pTRE2 expression vector (Clontech) containing RORα or RORγ into CHO Tet-on cell line (Clontech) and subsequently with pGL4.27 luciferase reporter vector (Promega) driven by 5xRORE. pGL4-27-5xRORE and pTRE2-ROR expressing cells were selected in medium containing puromycin (Sigma-Aldrich) and hygromycin (Invitrogen). CHO Tet-on cell lines were cultured in F12 medium supplemented with 10% FBS approved for the use in the Tet-on system (Clontech). To induce ROR expression cells were treated with 1 M doxycycline and a diluted series of compound inhibitors for 20 hr. RORE-mediated activation of the luciferase reporter was measured with a Luciferase Assay Substrate kit (Promega). Assays were performed in triplicate. cAMP-based cell viability was evaluated by CellTiter-Glo^®^ Luminescent Cell Viability Assay (Promega). To measure the activation of the *IL17* promoter, human T lymphocyte Jurkat cells were co-transfected with pCMV-β-Gal, pCMV10-3xFlag-RORγ, and a pGL4.14 reporter plasmid (Promega) containing human *IL17*-3kb-CNS promoter [20] using Lipofectamine 2000 (Invitrogen). To determine that the antagonist inhibited RORγ activity acted through the ligand binding domain (LBD) of RORγ, mammalian mono-hybrid analysis was performed. CHO cells were co-transfected with a pGL4.27-(UAS)_5_ reporter plasmid, pCMV-β-Gal, and pM-RORLBD [21]. To examine the interaction of the ROR activation domain with LXXLL-type co-activator motifs, we performed mammalian two-hybrid analysis. CHO cells were co-transfected with a pGL4.27-(UAS)_5_ reporter plasmid, pCMV-β-Gal, pM-EBIP96 containing the EBIP96 LXXLL co-activator motif fused to the Gal4(DBD), and VP16-RORγ(LBD) [21]. After 24 hr incubation, the luciferase and β-galactosidase activities were measured by Luciferase Assay Substrate kit (Promega) and Luminescent β-galactosidase Detection Kit II (Clontech). All transfections were performed in triplicate and repeated at least twice.

### Quantitative gene expression analysis of Jurkat clones

Jurkat cells were transfected with either pCMV10-3xFlag-RORγ expression vector or the empty vector using Lipofectamine 2000 (Invitrogen) and 24 hr later stimulated by 500 ng/ml phorbol 12-myristate 13-acetate (PMA) and 1 μM calcium ionophore A23187 (Sigma-Aldrich). Cells were lysed 5 hr later in RLT buffer and RNA was extracted using a QIAshredder column followed by RNeasy Mini kit (Qiagen, Valencia, CA) according to the manufacturer’s instructions. The RNA was reverse-transcribed using High-Capacity cDNA Archive Kit (Applied Biosystems). Gene expression analysis was performed by qRT-PCR analysis with SYBR Green I (Applied Biosystems, Foster City, CA). The reactions were carried out in triplicate in a 7300 Real Time PCR system (Applied Biosystems) using 20 ng of cDNA and the following conditions: 2 min at 45°C and 10 min at 95°C, followed by 40 cycles of 15 sec at 95°C and 60 sec at 60°C. All the results were normalized to the amount of *Gapdh* mRNA. Products specificity was routinely confirmed by melting curve analysis. qRT-PCR primer sequences are listed in S1 Appendix.

### Chromatin co-immunoprecipitation assay (ChIP)

The ChIP assay was performed using a ChIP assay kit from Millipore (Billerica, MA) according to the manufacturer’s protocol with minor modifications as described previously [22]. In short, Jurkat cells transfected with pCMV10-3xFlag-RORγ expression vector and stimulated by PMA-calcium ionophore were crosslinked by 1% formaldehyde for 10 min at room temperature. After a wash in PBS, the crosslinked chromatin was sonicated and incubated for 1 hr with ANTI-FLAG M2 Affinity Gel (Sigma-Aldrich). DNA-protein complexes were eluted by 3XFLAG Peptide (Sigma-Aldrich). The crosslinks were reversed by overnight incubation at 65°C in the presence of 25 mM NaCl, digested with RNase A and proteinase K, and then the ChIPed-DNA was purified. The amount of the ChIPed-DNA relative to each input DNA was determined by qPCR. All qPCR reactions were carried out in triplicate. Sequences of primers for ChIP-qPCR are listed in S1 Appendix.

### Human peripheral blood CD4+ T cell cultures and cytokine analysis

Cryopreserved human CD4+ T cells (AllCells, LLC and Stemcell Technologies, Inc.) were differentiated to the Th17 subtype by culturing for 5 days in CD3-coated tissue culture plates (2 μg/mL) in Iscove’s modified Dulbecco’s medium (IMDM) containing 10% HI-FBS, 55 μM 2-mercaptoethanol and soluble anti-CD28 (3 μg/mL) in the presence of a Th17 skewing cocktail, [IL-1β (10 ng/mL), IL-6 (30 ng/mL), TGFβ (0.5 ng/mL), IL-21 (10 ng/mL), IL-23 (10 ng/mL), anti-IFNγ (10 μg/mL) and anti-IL-4 (10 μg/mL)] in the presence or absence of serially diluted compounds. Secreted IL-17A and IL-22 protein was analyzed by MSD electrochemiluminescent cytokine assays (Mesoscale Discovery) and ELISA (Quantikine assay, R&D Systems), respectively. Message expression was assessed following total RNA isolation from CD4+ and Th17 polarized cells (pooled triplicate treatment wells) using the SV 96 total RNA isolation kit, and the reverse transcription into cDNA with the High capacity cDNA reverse transcription kit. cDNA aliquots were amplified over 40 cycles by real-time qPCR (Taqman) using a commercially available reaction master mix and FAM/MGB-labeled primer/ probe mixes for human *il17a*, *il17f* and *il22* transcripts with normalization to the housekeeping gene, cyclophillin B (PPIB).

### BioMAP® Diversity Plus System™

The BioMAP® Diversity Plus System™ includes 12 specific combinations of human cells stimulated to represent various disease conditions. This system also has the capacity to search for similar patterns of biological responses across the BioMAP reference database of >3000 compounds, biologics and approved drugs and experimental agents. Primary human cell types used in BioMAP systems and their stimuli include the following: 3C (umbilical vein endothelial cells (HuVEC)/IL-1β, TNFα and IFNγ), 4H (HuVEC/IL-4 and histamine), LPS (PBMC and HuVEC/LPS), Sag (PBMC and HuVEC/TCR ligands), BT (CD19+B cells and PBMC/anti-IgM + TCR ligands), BE3C (bronchial epithelial cells/ IL-1β, TNFα and IFNγ), BF4T (bronchial epithelial cells and human dermal fibroblasts/TNFα and IL-4), HDF3CGF (human dermal fibroblasts/ IL-1β, TNFα, IFNγ, EGF, bFGF and PDGF-BB), KF3CT (keratinocytes and dermal fibroblasts/ IL-1β, TNFα and IFNγ), CASM3C (coronary artery smooth muscle cells/ IL-1β, TNFα and IFNγ), MyoF (differentiated lung myofibroblasts/TNFα and TGFβ), /Mphg (HuVEC and M1 macrophages/TLR2 ligands (low hydrocortisone). Adherent cells are incubated to confluence at 37°C. Compounds are then added for 1 hr followed by addition of appropriate stimuli. Assay plates are then incubated for 24 hr for standard readouts. MyoF system is stimulated for 48 hr and BT is stimulated for either 72 hr (soluble readouts) or 6 d (secreted IgG). Cell proliferation is determined using either sulforhodamine B (SRB) assay for adherent cell types or Alamar Blue for PBMC cells. For proliferation assays, individual cell types are cultured at subconfluence and are read at specific times for different primary cell types (48, 72, or 96 hr). After stimulation, plates and supernatants are harvested and biomarkers quantified by ELISA.

### Imiquimod mouse studies

BALB/c JByRj Female Mice (8 week-old at study initiation) were treated with imiquimod cream (5%) or petrolatum (non-inflammatory inert cream), as described in the text. For three days of pre-treatment, and then daily until the end of the study (two hours before each application of IMQ), the same skin area was treated topically with compound in a simple ethanolic solution (60% ethanol: 40% water) at 1% and 0.1% or formulated in ointment at 1%. Clobetasol, a potent immonusupressor, was included as a positive control. Skin from the treatment area was harvested at day 3 or day 9 of IMQ treatment and RNA was isolated for gene expression by qPCR using the following TaqMan probes: *il17A* Mm00439618_m1; *il17F* Mm00521423_m1; *il22* Mm01226722_g1; *mip3α* or *ccl20* Mm01268754_m1; *kc* or *cxcl1* Mm04207460_m1; *tnf* Mm00443260_g1; *il1β* Mm00434228_m1; *il19* Mm01288324_m1; *ifnγ* Mm01168134_m1; *ip10* or *cxcl10* Mm00445235_m1; *actin b* Mm00607939_s1.

### Skin Resident Immune Cell Activation (sRICA) Assay

Full-thickness human skin was obtained within several hours from patients undergoing abdominoplasty from elective surgery. Immediately after collection, the skin was transferred to a plastic container with Phosphate Buffered Saline (PBS) and kept at 4°C during shipment and storage. Following shipment, human skin was defatted and dermatomed to 750 μm. The skin section was cut to 10 mm diameter round sections, and placed in the upper chamber of 0.4 um PCF membrane transwells (Millicell #PIHP01250) containing 30 μl of a 64% bovine collagen solution (Organogenesis, #200–055) resting in 6-well tissue culture plates. The tissue was supplemented with Cornification media (DMEM/F12, 90mM Adenine, 0.94M CaCl, 10nM Tri-iodothyronine, 1X ITS-X (Gibco 15100056), 100x Antibiotic/Antimycotic (Gibco 15240062), 2% heat-inactivated FBS, 1x Glutamax (Gibco 35050061), 0.01 mg/ml Gentamicin (Invitrogen #15750060); and maintained in a humidified incubator at 37°C for the duration of the study. Skin-resident immune cells were activated *in situ* under different conditions, including TCR alone [CD3 (1ug/ml), CD28 (2ug/ml)]; Th17 polarizing cocktail [TCR plus anti-IL-4 (1 ug/ml), anti-INFγ (1 ug/ml), IL-1β (10 ng/ml), IL-6 (10 ng/ml), TGFβ (1 ng/ml), IL-21 (10 ng/ml)]; or recombinant cytokines [rhIL-17 (10ng/ml R&Dsystems 317-ILB), rhIL-22 (10ng/ml R&Dsystems 782-IL/CF)], in the presence or absence of compound. For gene expression analysis, skin tissue was minced and homogenized in Precellys Homogenizer. mRNA was extracted using Qiagen RNeasy kit (Qiagen 74106) and quantified by NanoDrop. Samples were normalized to the lowest concentration sample and cDNA was made using Superscript Vilo cDNA synthesis Master Mix (Invitrogen 11755250). cDNA was used to perform qPCR on Applied Biosystems Viia7 System using Taqman Fast Advanced Master Mix (Invitrogen 4444965). mRNA levels of each gene of interest relative expression were calculated using the ΔΔCT formula. For topical compound application: Human skin explants (12mm biopsies) were obtained from abdominal skin and placed in 7 mm Franz Cell chambers supplemented with Cornification medium. Topical formulations containing vehicle or compound (8.4ul per sample) were applied to the skin using a positive displacement pipettor. After 24 hr media was changed to Th17 polarizing conditions, as defined above, and sections were harvested for gene expression analysis 24 hr later. Taqman Probes purchased from Applied Biosystem were as follows: GAPDH (Hs02758991_g1), IL17A (Hs00174383_m1), DEFB4 (Hs00823638_m1), IL1F9(IL-36G) (Hs00219742_m1), IL-19 (Hs00604657_m1), S100A7A (Hs00752780_s1), IL-23Ap19 (Hs00900828_g1), SERPINB4 (Hs01691258_g1), TGFa (Hs00608187_m1), S100A12 (Hs00942835_g1), IL1F8(IL-36B) (Hs00758166_m1), CEBPA (Hs00269972_s1), IL-1b(IL1F2) (Hs01555410_m1), CXCL10 (Hs01124251_g1), IL-17F (Hs00369400_m1) and Il-22 (Hs01574154_m1).

### Histology (H&E staining)

Biopsy sections were fixed in 10% neutral buffered formalin for 72 hours. Cassettes containing biopsies were processed on a Microm STP120 using a 5½ hour schedule without heat or vacuum, embedded in paraffin, sectioned at 4 μm and mounted on charged slides. Sections were air dried at room temperature overnight, then stained with hematoxylin & eosin (H&E) as follows: deparaffinize slides and rehydrate through a series of alcohols to water, stain in Richard Allen Hematoxlyin 2 (ThermoFisher REF7231) for 2 minutes and in phloxine-eosin (ThermoFisher REF71304) for 22 seconds, dehydrate to xylene, and coverslipped. Slides were analyzed and representative pictures for each treatment group were taken using a Nikon Eclipse 600 with a 40x objective.

## Results

Development of an effective topical medicine requires, (i) identifying a new chemical entity (NCE) that impacts on the desired biological target (ii) demonstration that the NCE engages its target and can revert disease phenotype, and (iii) has a safety profile commensurate with its anticipated use in the human population. Several groups have identified synthetic ligands that target RORγt via distinct mechanisms, as recently summarized [23]. For instance, ligands can induce a conformational change that, (i) reduce coactivator protein recruitment (inverse agonists), (ii) promote coactivator protein recruitment (agonist), or (iii) do not affect basal transcriptional activity (silent ligands/neutral antagonists). Examples of RORγ synthetic ligands and the relationship between ligand structure and their specific mechanisms of action have been described for the tertiary sulfonamide ligand class [23,24]. Treatment with these inverse agonists lead to reduced IL-17 production, suggesting that such compounds could be considered for therapeutic development.

### 1. Establishing compound mechanism of action (MOA)

Towards the goal of developing a topical medicine to disrupt IL-17-driven skin inflammation, we examined three novel small molecules (GSK compounds 2981278, 3038548 and 3038549). Upon initial profiling, all three compounds reduced basal RORγ transcription, consistent with inverse agonist activity (data not shown).

**Fig 1:**
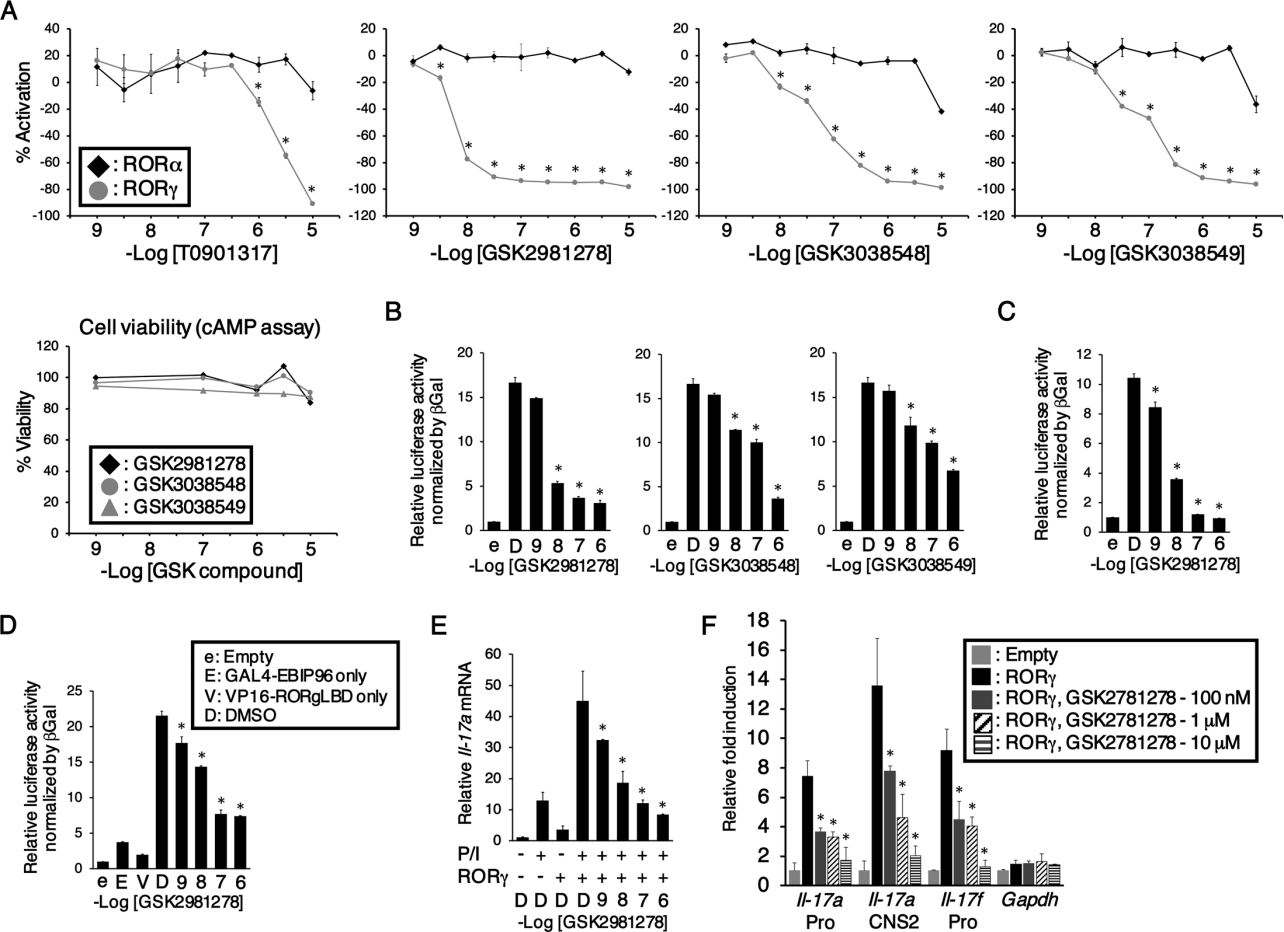
GSK2981278 is a strong RORγ-selective inverse agonist that inhibits activation of the *il17* promoter. (A) RORE-dependent activation was examined using a Luciferase-based reporter (pGL4-27-5xRORE) CHO Tet-on cell line. Percent inhibition was calculated relative to vehicle (DMSO). cAMP-based cell viability was measured. (B) Activation of the human *il17* promoter in compound-treated Jurkat cells co-transfected with control or reporter plasmid. D, indicates DMSO. (C-D) Mammalian mono-hybrid (C) or two-hybrid (D) analysis was performed following co-transfection with control or reporter plasmids +/**-** compound. (E) pCMV10-3xFlag-RORγ-transfected Jurkat cells were stimulated for 24 hrs and *il17* mRNA levels determined by qRT-PCR. (F) ChIP-qPCR was performed in pCMV10-3xFlag-RORγ-transfected Jurkat cells. All experiments were independently performed at least twice. Significant inhibition was determined by Student’s *t* test. (* p≤0.01).

We next assessed the potency and selectivity of each compound in *in vitro* reporter assays. First, the effect of these compounds on RORα and RORγt transcriptional activity was evaluated in doxycycline-inducible CHO stable cell lines (Fig 1A). T0901317, a well-described ROR inhibitor [25], reduced the RORE-dependent activation of a luciferase reporter at concentrations of 1 μM-10 μM. GSK2981278 was the most effective of those tested in reducing RORγt-reporter gene activation. Further, GSK2981278 had no significant effect on RORα-dependent activation, indicating its selectivity for RORγ. GSK3038548 and GSK3038549 also inhibited RORγt transactivation, although not as effectively as GSK2981278. None of the three RORγ inverse agonist compounds demonstrated significant activity against RORα, nor did they induce cell death at concentrations up to 10 μM. In addition to reducing activity of the synthetic RORE promoter in CHO cells, all three compounds reduced RORγt-dependent activation of the *il17* promoter, as shown using an immortilized T cell-based reporter assay (Fig 1B). In recent years, several RORγ inhibitors have been reported in the literature [26–30]. These compounds demonstrate potency of 0.01 and 0.04 μM in luciferase-based reporter assays, which is on par with previously reported RORγ inverse agonists (SR2211 or digoxin) [31,32]. Importantly, GSK2981278 demonstrates 10-fold greater potency for IL-17A inhibition than T0901317, GSK3038548, GSK3038549 or many of those recently described [31,32]. As such, further investigation focused primarily on GSK2981278. GSK2981278 inhibited RORγt-mediated transactivation in mammalian mono-hybrid analyses (Fig 1C). That GSK2981278 inhibits the interaction, and possibly recruitment of RORγ with co-activators is indicated by inhibition of the interaction of RORγ with the co-activator peptide EBIP96 in two-hybrid analysis (Fig 1D). Next, we examined the effect of GSK2981278 on endogenous *il17a* expression in Jurkat cells. GSK2981278 repressed the activation of *il17a* by RORγt in a dose-responsive manner (Fig 1E). With chromatin immunoprecipitation (ChIP), we show that GSK2981278 attenuates the recruitment of RORγ to endogenous *il17* promoter regions (Fig 1F). RORγt inhibitors can modulate cytokine transcription via distinct mechanisms [28]: (i) inhibition of IL-17A transcriptional activity by interfering with RORγ-DNA binding, and (ii) reduced transcriptional activity due to compound-dependent differences in the co-activator/co-repressor interactions of RORγ. These data identify the mechanism of GSK2981278 as one that interferes with RORγ-DNA binding because we observed a reduction in the amount of RORγt bound to the *il17a* promoter region, in addition to inhibiting the interaction with co-activator peptides.

### 2. Biological activity: confirmation of target engagement in human peripheral T cell assays

Next, we measured the impact of GSK2981278 on human peripheral blood CD4+ T cell cytokine production. During 5 days of culture under Th17 skewing conditions, IL-17A and IL-22 protein secretion was markedly and potently inhibited by GSK2981278 in a concentration dependent manner (IC_50_ = 3.2 nM) (S3 Appendix). Culture in the presence of ≥3 nM GSK2981278 led to a near-complete inhibition of IL-17A protein secretion. In contrast, IL-22 cytokine levels were reduced by only 50–70%, indicative of a more complex regulatory process for IL-22 expression. Indeed, it is now well acknowledged that IL-22 is produced by a subset of Th17 cells as well as the distinct Th22 T cell subpopulation. As such, our results likely represent the combined secretion of Th17 and Th22 cells that may be present in our culture system, which may not be completely abrogated by RORγ inhibition alone.

GSK2981278 robustly inhibits RORγ-mediated cytokine production at both the mRNA and protein level. Consistent with the effects on protein, expression of cytokines *il17a*, *il17f* and *il22* was reduced ≥50% relative to vehicle-treated control cultures (S3 Appendix). Intriguingly, significant decreases in expression levels were observed at concentrations as low as 30 pM.

### 3. Compound Specificity: BioMAP Profile of GSK2981278 demonstrates selectivity for IL-17 family cytokines

In drug development, off target effects can often limit the practical utility of a new medicine. Therefore, we predicted the *in vivo* signature after exposure to GSK2981278 using a platform of primary human cell-based assays, referred to as the BioMAP® Diversity Plus System™. The BioMAP system includes 12 specific combinations of primary human cells (endothelial cells, peripheral blood mononuclear cells, B cells, epithelial cells, T cells, macrophages, fibroblasts, keratinocytes and smooth muscle cells) stimulated to activate multiple disease-relevant signaling pathways [33]. This systems allows profiling the biologic effects of compounds across a panel of disease-relevant biomarkers. In addition, this system can compare patterns of biological responses across the BioMAP reference database of >3000 compounds, biologics and approved drugs [34]. Thus, BioMAP® can identify common activity profiles, which may translate to similar mechanisms of action between distinct classes of inhibitors. According to this platform, GSK2981278 selectively inhibited only 2 specific biomarkers: IL-17A and IL-17F (S3 Appendix). Further, the profile of GSK2981278 did not significantly align with any other compound in the BioMAP reference database (Pearson’s correlation threshold ≥0.7), highlighting the selectivity of the compound for RORγt-dependent cytokines and the low probability of significant interactions with secondary targets.

### 4. Biologic activity: *In vivo* efficacy, mouse model

**Fig 2:**
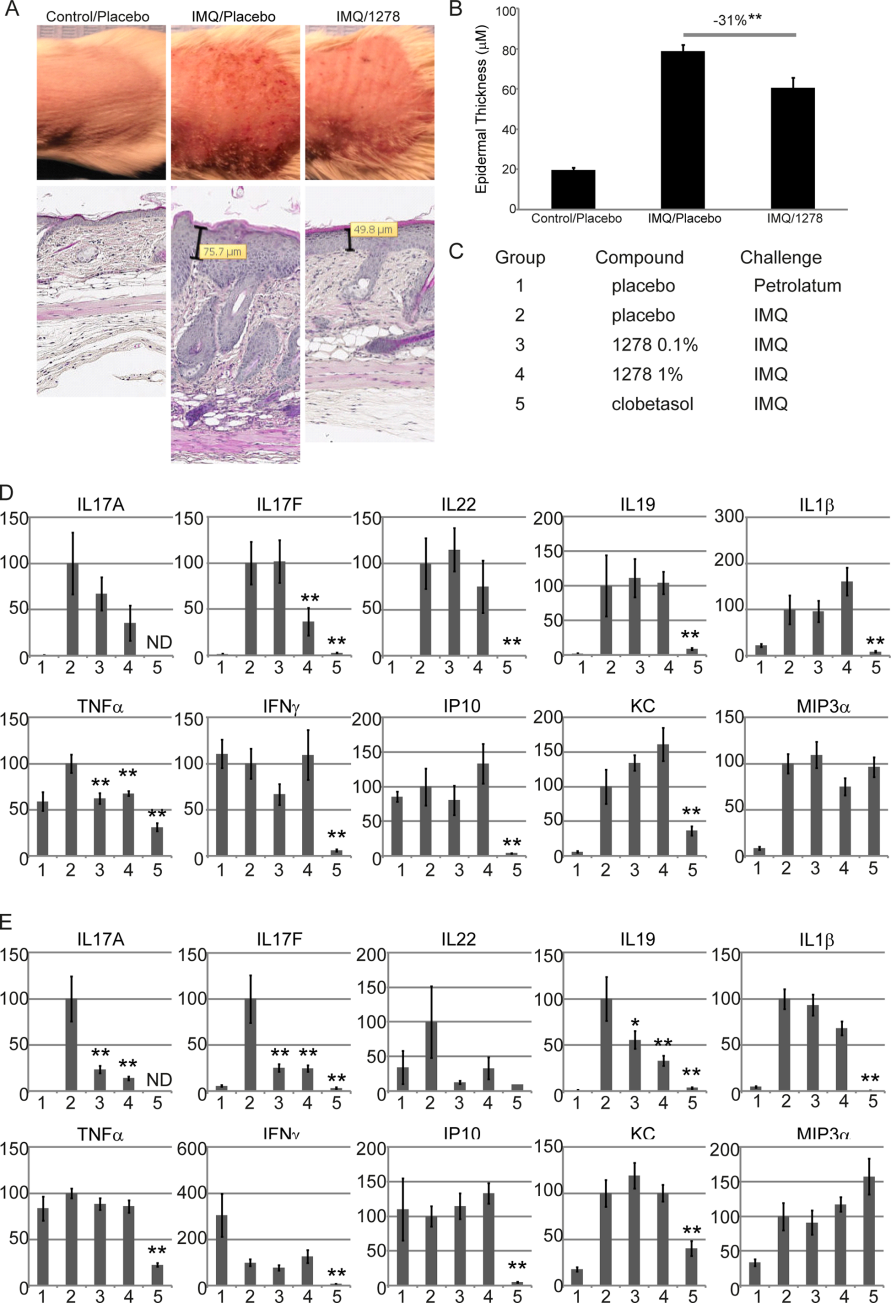
GSK2981278 attenuates inflammation in a mouse model of psoriasis. (A) Mice were treated topically with placebo or 1% GSK2981278 (1278) in ointment, and with imiquimod (IMQ) or petrolatum (vehicle). At study’s end (day +9 of IMQ treatment), back skin was imaged and stained (H&E). (B) Mean epidermal thickness is shown across 6–9 mice per treatment group. (C-E) Changes to local cytokine expression was determined following topical application of 1% or 0.1% compound in a simple ethanolic solution (60:40 ethanol:water). (C) Description of study groups for panels D-E. Skin cytokine levels on day +3 (D) or day +9 (E). Data reflect the mean ± SEM gene expression level across 6–9 mice per treatment group. Significant inhibition was determined by Student’s *t* test. (*p≤0.05;**p≤0.01).

Although our *in vitro* studies demonstrate the ability to inhibit IL-17 production, *in vitro* assays may not always predict a compound’s behaviour in complex *in vivo* systems. The imiquimod (IMQ) mouse model was employed to demonstrate efficacy of GSK2981278 in a psoriaform-like mouse model. Topical application of IMQ can induce and exacerbate psoriasis-like chronic skin inflammation in mice, including epidermal thickening, a dependence on T cell immunity, and a mechanism dependent on the IL-23/IL-17 pathway [35]. We first confirmed GSK2981278 can inhibit IL-17A protein secretion by mouse CD4+ T cells, confirming cross-reactivity of our compound for both mouse and human RORγ (data not shown). Next, mice were treated topically with GSK2981278 (1% ointment or placebo) for three days (day -3 to day 0), after which mice continued to receive compound for the duration of the study. Starting on day 0, mice were challenged topically with IMQ (5%) cream or petrolatum (non-inflammatory inert cream) for up to ten days (day 0 to day +9). On the last day of treatment, the skin was imaged and clinically assessed. GSK2981278 was undetectable (<5 ng/ml) in serum at harvest, indicating that systemic exposure was minimal. Mice exposed to GSK2981278 exhibited reduced skin redness and scaling, as well as decreased hyperplasia, as evidenced by a 23% reduction in epidermal thickness when compared to the placebo + IMQ-treated group (Fig 2A and 2B).

We next compared the specificity of GSK2981278 to the broad spectrum immunomodulatory agent clobetasol. Mice were treated with GSK2981278 (1% or 0.1% in ethanolic solution) or clobetasol, a class I ultrapotent topical corticosteroid, daily for three days of pre-treatment and then daily for the duration of the study, as described above. The study cohorts and their respective treatments are defined in Fig 2C. Cytokine expression in treated skin was determined at either day +3 or day +9 post challenge by qPCR. Indeed, a dose-dependent effect was observed for *il-17a* and *il-17f* at 3 days post-IMQ challenge. In contrast, the levels of other cytokines tested were not impacted at this early timepoint (Fig 2D). By day +9, *il17a*, *il17f*, and *il22*, were dramatically inhibited by treatment at both concentrations, whereas the expression of *il19* and, less so, *il1β*was inhibited in a dose-dependent manner (Fig 2E). Importantly, IL-19 is expressed by keratinocytes following stimulation with IL-17A [36], demonstrating that the effects of GSK2981278 are cytokine-specific. In contrast, clobetasol treatment inhibited nearly all cytokines non-specifically, including *il1β*, *il19*, *IP10*, *KC* and *ifnγ* (Fig 2D and 2E).

### 5. Biologic activity/target engagement: Development of a novel *ex vivo* human skin model for IL-17-driven skin inflammation

**Fig 3:**
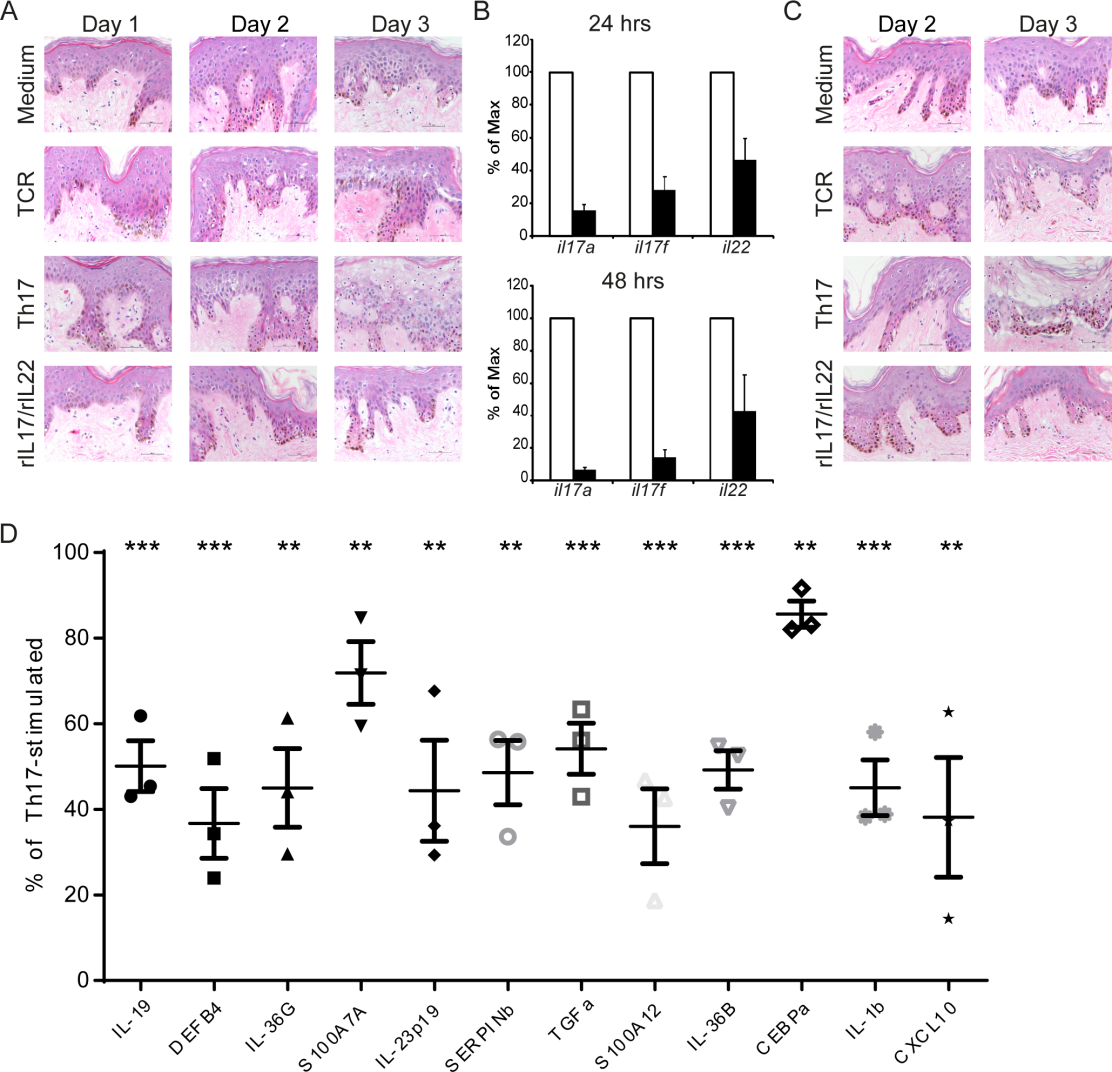
Skin Resident Immune Cell Activation (sRICA) leads to pro-inflammatory cytokine responses that are reduced by GSK2981278. (A) Skin explants were cultured ≥4 days under the indicated conditions. Explants were analyzed for tissue integrity by H&E. (B) Samples were pre-treated with 10 μM compound (closed bar) or vehicle (DMSO; open bars–set to 100%) for 1 day prior to 24–48 hrs of Th17 stimulation. Relative transcript levels were determined by qRT-PCR. (C) Samples were treated as in B, then analyzed daily for tissue integrity by H&E. Images are representative at least 3 independent experiments. (D) Samples were treated as in B. Graphs show the mean percent maximum stimulation of 3 independent experiments. Significant inhibition was determined by Student’s *t* test. (*p≤0.05; **p≤0.01; ***p≤0.001).

While IMQ-treated mice recapitulate many properties of human psoriatic lesions, the model has limitations. For instance, the IMQ-mouse model cannot be used for target validation of species-specific human targets nor can it effectively validate topical treatments because of significant differences in the barrier properties of rodent and human skin. Therefore, we developed a novel tissue-based assay that exploits *in vitro* activation of skin-resident immunocompetent cells, termed skin-Resident Immune Cell Activation (sRICA) Assay, to evaluate the therapeutic potential of compounds for dermatological diseases. Activation of immunocompetent cells and subsequent release of cytokines are hallmark features of inflammation, and the *in vitro* activation of blood-derived leukocytes is a common laboratory procedure. Moreover, healthy human skin is enriched for a multitude of hematopoietic cell lineages, including Langerhan’s Cells, CD4+ and CD8+ T cells, γδT cells, various DC populations, and innate lymphoid cells (ILCs) [37,38]. Therefore, we reasoned that skin-resident immunocompetent cells could be activated *in situ* to induce specific cytokine/chemokine responses, analogous to blood derived leukocytes cultured under specific polarizing conditions. Skin-resident immune cells were activated under Th17-skewing conditions to mimic the predominately Th17-polarized profile of lesional psoriatic skin. Importantly, excised healthy human skin remains viable in culture for greater than 1 week based on the structure and integrity of the epidermis and underlying dermis (data not shown). However, prolonged stimulation in the sRICA Assay led to epidermal cell death characterized by a swelling of the cytoplasm (termed ballooning degeneration) and clefting of the epidermis away from the dermis (Fig 3A). We determined that accelerated degradation of the tissue was a by-product of Th17-skewed proinflammatory cytokine expression because T cell activation alone (CD3/CD28 crosslinking in the absence of skewing conditions) led to significantly less ballooning degeneration than that observed in Th17-skewed cultures. Likewise, skin cultured only with recombinant cytokines (rhIL-17A and rhIL-22) exhibited a decline in epidermal viability similar to that of Th17-skewing conditions (Fig 3A). Nonetheless, these data confirm that tissue integrity is maintained for more than 48 hours post stimulation; thereby, providing a 2-day window within which immunomodulatory compounds can be tested.

Message expression of *il17a*, *il17f* and *il22* was used to evaluate transcriptional activity of RORγt. First, we confirmed that RORγt-dependent transcription was present throughout the sRICA Assay based on the level of *il17a* mRNA isolated from *ex vivo* human skin tissue (data not shown) and validated that sRICA-induced cytokine expression could be reduced in the presence of RORγt inhibitor compounds, including digoxin-derivatives [31] and compounds from our small molecule series (S4 Appendix). Next, we examined whether GSK2981278 impacted cytokines; *Il17a*, *il17f* and *il22* transcript levels were dramatically abrogated at 24 and 48 hrs post stimulation (Fig 3B). While pretreatment of healthy skin tissue with GSK2981278 alone did not negatively impact tissue viability or accelerate the damaging effects of sRICA, it did not protect against activation-induced tissue damage at 3 days post stimulation, in spite of nearly 90% inhibition of *il17a* message expression (Fig 3C). Because keratinocyte responses and epidermal changes are also hallmarks of inflammatory skin disease and because secondary activation of keratinocytes, including downstream cytokine and chemokine production, are likely products of the sRICA Assay, we evaluated a selection of RORγt-independent genes overexpressed in human psoriasis: *cxcl10*, *il1β*, *cebpa*, *il36b* (*il1f8*), *s100a12*, *tgfα*, *serpinb*, *il23p19*, *il23p40*, *s100a7a*, *il19*, *il36g* (*il1f9*), *defb4* [7]. Among them, *cxcl10*, *defensin B4* (*defB4*) and *il36g* (*il1f9*) transcript levels exhibited the greatest post-activation increase above naïve human tissue (220-, 120- and 112-fold increases at 48 hrs post activation, respectively). Expression of *il23p40* was significantly inhibited in the presence of GSK2981278 (82 ± 9% inhibition at 24 hours post-stimulation). Likewise, exposure to GSK2981278 significantly reduced the transcript levels of all cytokines tested at 48 hours post-stimulation (Fig 3D). These data support the notion that selective inhibition of RORγt impacts pro-inflammatory cytokine expression, including secondary gene activation induced in the sRICA Assay stimulated under Th17 conditions. These secondary responses include keratinocyte-derived factors that more broadly encompass the psoriasis signature, and these are also reduced when the pro-inflammatory IL-17 response is suppressed by RORγt inhibition. Notably, these factors are not completely abrogated in the presence of compound, which may explain why RORγ inhibition does not protect from activation-induced decreases in tissue viability.

### 6. Dermal penetration/topical target engagement: Topical formulation effectively delivers API through the stratum corneum to engage its target and disrupt IL-17a/f production

**Fig 4:**
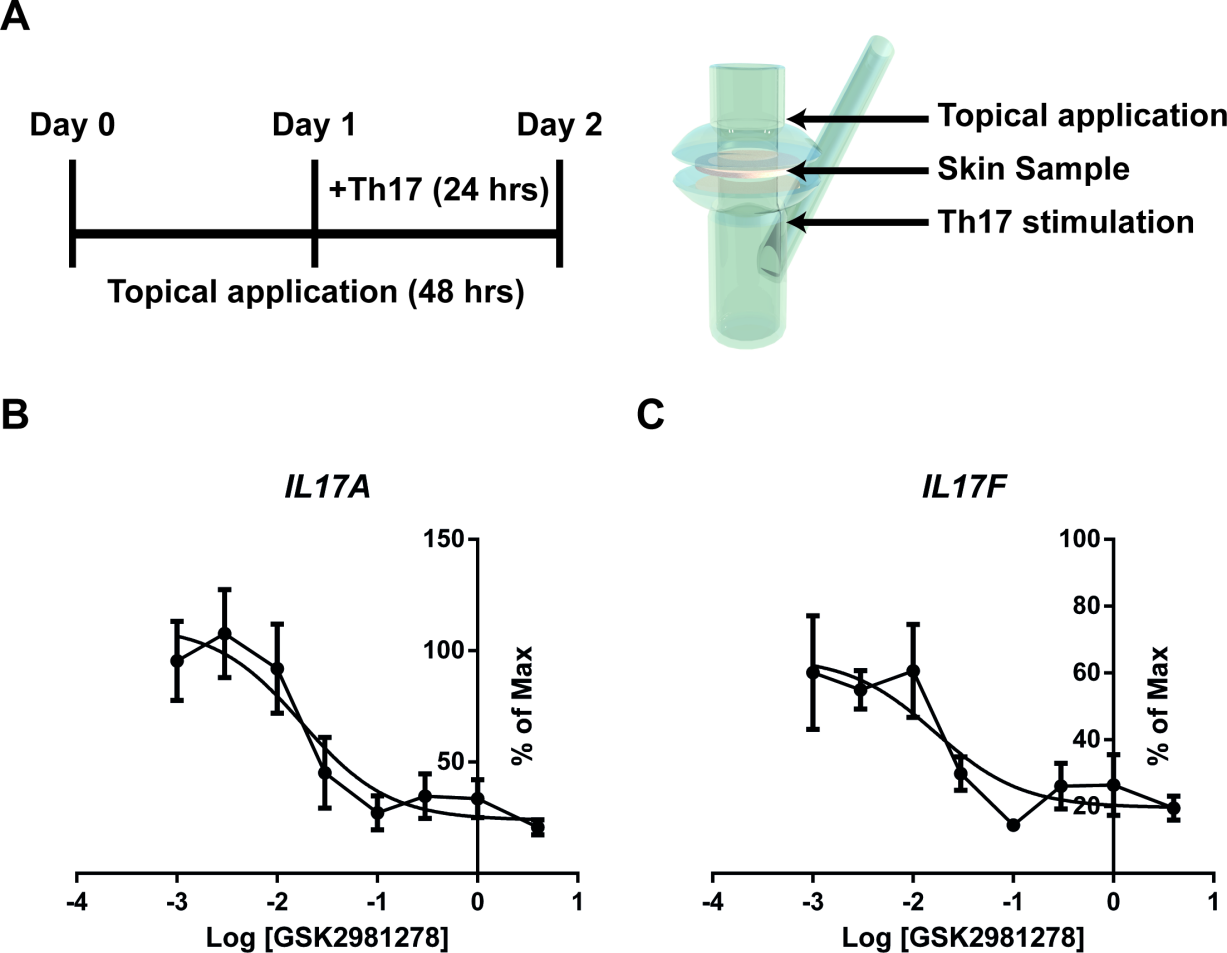
Suppression of Th17-type cytokine production following topical application. *Ex vivo* human skin was cultured in Franz Cell chambers for a total of 48 hours. GSK2981278 was applied to the dry surface of the skin at time zero followed 24 hrs later by activation of skin resident immune cells under Th17 polarizing conditions. The experimental schema is shown in panel A. Skin sections were harvested after 24 hrs of stimulation (48 hrs of compound treatment) and analyzed for relative gene expression of *il17a* (B) or *il17f* (C). Data are shown as the percent maximum expression of each cytokine as compared to Th17-stimulated samples treated topically with vehicle only.

Many dermal products are formulated as gels, creams or lotions for topical delivery. However, the interaction of a drug product with the various components of a complex formulation may impact its activity. Therefore, to confirm that GSK2981278 remains biologically active once formulated for topical delivery, we adapted the sRICA assay to customized Franz cells, which clamp the edges of the skin to prevent leakage of topical compounds into the lower chamber of the air-liquid interface culture. As such, delivery through the skin barrier and inhibition of the compound’s dermal target were examined in parallel. Skin biopsy sections were first exposed to GSK2981278 in a simple ointment at approximately half-log increments between 0.001% and 4% strengths. The ointment was then left undisturbed for the duration of the culture. Twenty-four hours after topical application of GSK2981278 in ointment, the media in the lower chamber was replaced with media containing Th17-skewing factors to activate skin-resident immune cells for an additional 24 hrs (Fig 4). Importantly, GSK2981278 potently and dose-dependently inhibited cytokines in this assay, with ~50% reduction of both *il17a* and *il17f* with the 0.02% formulation.

### 7. Efficacy on human disease tissue: psoriatic signature genes are reduced in lesional psoriatic skin following GSK2981278 treatment

**Fig 5:**
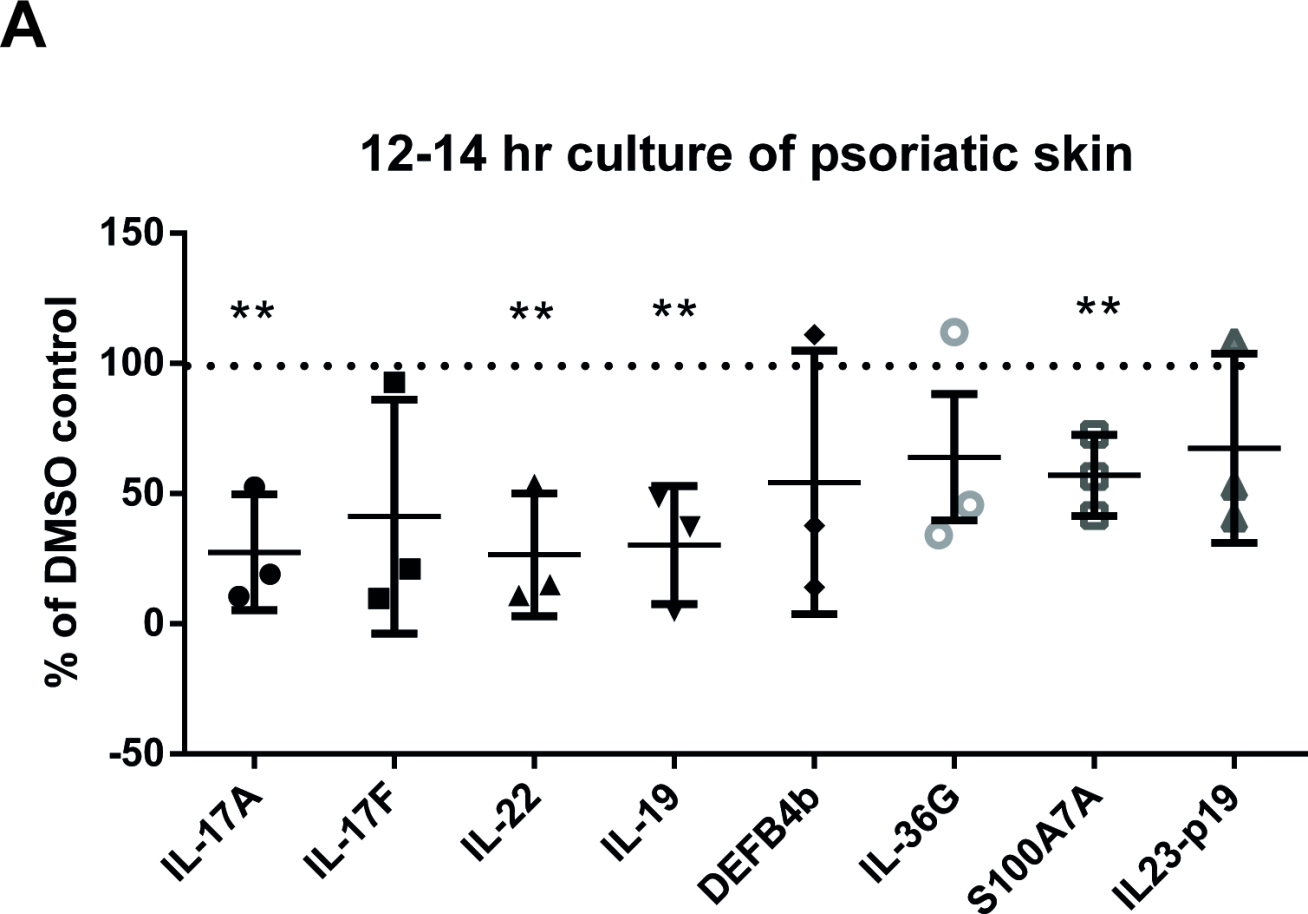
Treatment of psoriatic tissue with the RORγ inverse agonist GSK2981278 reduces proinflammatory cytokine levels. Three psoriatic skin biopsies were obtained via 3-4mm punch biopsy and placed in Unisol buffer for overnight shipment. Upon arrival, biopsy sections were placed in Cornification media without stimulation for 12–14 hours with either 0.2% DMSO or 10 μM compound. The percent inhibition of each biomarker compared to culture with DMSO alone is shown. Each point represents an individual patient sample. The percent maximum expression ± SEM for each analyte assayed of each tissue was calculated relative to time zero. Significant inhibition was determined by Paired *t*-test. (**p≤0.01).

The sRICA Assay is an efficient and reliable surrogate for acute skin inflammation with which immunomodulatory compounds can be tested. Nonetheless, it does not fully recapitulate the human disease state. Therefore, to test whether GSK2981278 could impact IL-17 cytokine production in diseased skin, we obtained lesional biopsies from psoriasis patients and evaluated whether exposure to GSK2981278 *ex vivo* could reduce proinflammatory cytokine levels. Two 3-mm biopsy sections per patient were obtained from the same psoriatic plaque. These sections were then bisected to yield 2 timepoints each: (i) time zero, representing the basal levels for that particular biopsy sample, and (ii) after ~12 hrs of culture with either vehicle (DMSO) or 10 μM GSK2981278. After treatment with compound, *il17a*, *il17f*, *il22* and *il19* transcript levels were reduced ≥50% compared to vehicle-treated psoriatic explants (Fig 5). In addition, *DefB4b*, *il36g*, *s100a7a* and *il-23p19* transcript levels were reduced to varying degrees, suggesting that the downstream effects of proinflammatory cytokines on keratinocyte gene expression are inhibited in psoriatic skin analogous to that seen in the sRICA Assay (Fig 5). Taken together with the sRICA Assay results, these data demonstrate that topical formulation of GSK2981278 can penetrate the skin barrier and reduce biomarkers of skin inflammation, even in diseased tissue. Together, these data provide the rationale to progress the clinical development of GSK2981278 as a topical medicine for psoriasis.

## Discussion

The pathophysiology of psoriasis involves signature Th17 cytokines, suggesting that targeting RORγt could prove an effective therapy for patients. In this report, we show that the compound GSK2981278 is a highly potent and selective inverse agonist for RORγ that inhibits production of the Th17 signature cytokines in multiple *in vitro* cell-based assays, *ex vivo* human models and in an *in vivo* mouse model of psoriasis. To investigate the effectiveness of GSK2981278 on inflammatory skin diseases, we developed the novel tissue-based sRICA assay, in which skin resident immune cells are activated *in situ* to mimic a localized inflammatory response. Importantly, with different stimulatory conditions, a wide variety of inflammatory processes can be modeled in this system, including Th1, Th2 and Th17 polarized immune responses. The sRICA Assay system allows for validation of target engagement of NCEs in a human system prior to clinical investigation. By adapting the sRICA assay for topical delivery, we demonstrate simultaneous penetration of the skin barrier and dermal target engagement. Further efforts are underway to quantify drug levels in the skin during sRICA target engagement studies by adapting this model to interrogate tissue-specific PK/PD relationships (Bedard M. et al., manuscript in preparation). Finally, while other RORγ inverse agonists have been used systemically to modulate cytokine expression in the IMQ mouse model or *in vitro* with isolated mononuclear cells from psoriasis patients [30], this report is the first demonstration of *in vivo* and *ex vivo* efficacy of an RORγ inverse agonist delivered topically.

The pivotal role of systemic IL-17 and IL-23 levels in psoriasis has been validated clinically with neutralizing antibodies (e.g. Ixekizumab, Secukinumab, Guselkumab and Tildrakizumab). Whether inhibiting local cytokine production can deliver effective relief to patients has yet to be shown. Our data demonstrate that GSK2981278 significantly inhibits production of the Th17 signature (IL-17A, IL-17F, IL-22 and IL-23) in multiple *in vitro* and human tissue-based assays, including topical delivery of the compound *via* the sRICA Assay and psoriatic lesional explants. As such, we hypothesize that topical delivery of this RORγ-specific inverse agonist will impact the local expression of cytokines while minimizing systemic bioavailability and potential toxicity concerns that may arise from systemic exposure. If tissue cytokine production is the main driver of inflammation in plaque psoriasis, our data suggest that topical treatment with GSK2981278 will significantly limit Th17-type cytokine expression and should therefore lead to improved clinical outcomes for patients. As such, these data support the progression of GSK2981278 to the clinic, a key milestone in the drug development process.

## Supporting Information

S1 Appendix Sequences of primers used for qPCR and ChIP-qPCR in Fig 1. (DOCX)

S2 Appendix Readout parameters used for BioMAP^®^ in S3 Appendix (DOCX)

S3 Appendix GSK2981278 potently and selectively inhibits IL-17 and IL-22 levels. (DOCX)

S4 Appendix RORγ inverse agonist reduces sRICA-induced *il17a* transcript levels in ex vivo human skin. (DOCX)
