# Supplementary material for: Development of a Topical Treatment for Psoriasis Targeting RORγ: From Bench to Skin
Source: PLoS One. 2016 Feb 12;11(2):e0147979. doi: 10.1371/journal.pone.0147979 (PMC4752338; doi:10.1371/journal.pone.0147979)
Supplement: S1 Appendix — (DOCX) [file pone.0147979.s001.docx]

**S1 Appendix. Sequences of primers used for qPCR and ChIP-qPCR in Figure 1**

| **Gene** | | **Sense primer** | **Antisense primer** |
| --- | --- | --- | --- |
| *Il17a* | GGTCCTCAGATTACTACAACCGATC | | GGATATCTCTCAGGGTCCTCATTG |
| *Gapdh* | CCCATCACCATCTTCCAGGAG | | CTTCTCCATGGTGGTGAAGACG |
| *Il17a* Pro | TGTGCCTGCTATGAGATGGAC | | CACGTAAGTGACCACAGAAGGAG |
| *Il17a* CNS2 | GTCAGCATTACCAAAGGGAAGTC | | ACCTATACGTTAGCAGGCACATG |
| *Il17f* Pro | GTAACTCCGGGTCAACCACAAC | | GTATCTTCCTGTGTATGCCTGTGTTC |
| *Gapdh* negative | TCCCTTGGGTATATGGTAACCTTG | | CCACTTGATTTGGAGGGATCTC |
