# Supplementary material for: Development of a Topical Treatment for Psoriasis Targeting RORγ: From Bench to Skin
Source: PLoS One. 2016 Feb 12;11(2):e0147979. doi: 10.1371/journal.pone.0147979 (PMC4752338; doi:10.1371/journal.pone.0147979)
Supplement: S2 Appendix — (DOCX) [file pone.0147979.s002.docx]

**S2 Appendix. Readout parameters used for BioMAP^®^ in S3 Appendix (listed in the order shown in S3 Appendix from left to right)**

| **SYSTEM NAME** | **READOUT PARAMETER** |
| --- | --- |
| 3C | CCL2/MCP-1 |
| 3C | CD106/VCAM-1 |
| 3C | CD141/Thrombomodulin |
| 3C | CD142/Tissue Factor |
| 3C | CD54/ICAM-1 |
| 3C | CD62E/E-Selectin |
| 3C | CD87/uPAR |
| 3C | CXCL8/IL-8 |
| 3C | CXCL9/MIG |
| 3C | HLA-DR |
| 3C | Proliferation |
| 3C | SRB |
| 4H | CCL2/MCP-1 |
| 4H | CCL26/Eotaxin-3 |
| 4H | CD106/VCAM-1 |
| 4H | CD62P/P-selectin |
| 4H | CD87/uPAR |
| 4H | SRB |
| 4H | VEGFR2 |
| LPS | CCL2/MCP-1 |
| LPS | CD106/VCAM-1 |
| LPS | CD141/Thrombomodulin |
| LPS | CD142/Tissue Factor |
| LPS | CD40 |
| LPS | CD62E/E-Selectin |
| LPS | CD69 |
| LPS | CXCL8/IL-8 |
| LPS | IL-1alpha |
| LPS | M-CSF |
| LPS | sPGE2 |
| LPS | SRB |
| LPS | sTNF-alpha |
| SAg | CCL2/MCP-1 |
| SAg | CD38 |
| SAg | CD40 |
| SAg | CD62E/E-Selectin |
| SAg | CD69 |
| SAg | CXCL8/IL-8 |
| SAg | CXCL9/MIG |
| SAg | PBMC Cytotoxicity |
| SAg | Proliferation |
| SAg | SRB |
| BT | B cell Proliferation |
| BT | PBMC Cytotoxicity |
| BT | Secreted IgG |
| BT | sIL-17A |
| BT | sIL-17F |
| BT | sIL-2 |
| BT | sIL-6 |
| BT | sTNF-alpha |
| BE3C | CD54/ICAM-1 |
| BE3C | CD87/uPAR |
| BE3C | CXCL10/IP-10 |
| BE3C | CXCL11/I-TAC |
| BE3C | CXCL8/IL-8 |
| BE3C | CXCL9/MIG |
| BE3C | EGFR |
| BE3C | HLA-DR |
| BE3C | IL-1alpha |
| BE3C | Keratin 8/18 |
| BE3C | MMP-1 |
| BE3C | MMP-9 |
| BE3C | PAI-I |
| BE3C | SRB |
| BE3C | tPA |
| BE3C | uPA |
| BF4T | CCL2/MCP-1 |
| BF4T | CCL26/Eotaxin-3 |
| BF4T | CD106/VCAM-1 |
| BF4T | CD54/ICAM-1 |
| BF4T | CD90 |
| BF4T | CXCL8/IL-8 |
| BF4T | IL-1alpha |
| BF4T | Keratin 8/18 |
| BF4T | MMP-1 |
| BF4T | MMP-3 |
| BF4T | MMP-9 |
| BF4T | PAI-I |
| BF4T | SRB |
| BF4T | tPA |
| BF4T | uPA |
| HDF3CGF | CCL2/MCP-1 |
| HDF3CGF | CD106/VCAM-1 |
| HDF3CGF | CD54/ICAM-1 |
| HDF3CGF | Collagen I |
| HDF3CGF | Collagen III |
| HDF3CGF | CXCL10/IP-10 |
| HDF3CGF | CXCL11/I-TAC |
| HDF3CGF | CXCL8/IL-8 |
| HDF3CGF | CXCL9/MIG |
| HDF3CGF | EGFR |
| HDF3CGF | M-CSF |
| HDF3CGF | MMP-1 |
| HDF3CGF | PAI-I |
| HDF3CGF | Proliferation_72hr |
| HDF3CGF | SRB |
| HDF3CGF | TIMP-1 |
| HDF3CGF | TIMP-2 |
| KF3CT | CCL2/MCP-1 |
| KF3CT | CD54/ICAM-1 |
| KF3CT | CXCL10/IP-10 |
| KF3CT | CXCL8/IL-8 |
| KF3CT | CXCL9/MIG |
| KF3CT | IL-1alpha |
| KF3CT | MMP-9 |
| KF3CT | PAI-I |
| KF3CT | SRB |
| KF3CT | TIMP-2 |
| KF3CT | uPA |
| CASM3C | CCL2/MCP-1 |
| CASM3C | CD106/VCAM-1 |
| CASM3C | CD141/Thrombomodulin |
| CASM3C | CD142/Tissue Factor |
| CASM3C | CD87/uPAR |
| CASM3C | CXCL8/IL-8 |
| CASM3C | CXCL9/MIG |
| CASM3C | HLA-DR |
| CASM3C | IL-6 |
| CASM3C | LDLR |
| CASM3C | M-CSF |
| CASM3C | PAI-I |
| CASM3C | Proliferation |
| CASM3C | Serum Amyloid A |
| CASM3C | SRB |
| MyoF | alpha-SM Actin |
| MyoF | bFGF |
| MyoF | CD106/VCAM-1 |
| MyoF | Collagen I |
| MyoF | Collagen III |
| MyoF | Collagen IV |
| MyoF | CXCL8/IL-8 |
| MyoF | Decorin |
| MyoF | MMP-1 |
| MyoF | PAI-I |
| MyoF | SRB |
| MyoF | TIMP-1 |
| Mphg | CCL2/MCP-1 |
| Mphg | CCL3/MIP-1alpha |
| Mphg | CD106/VCAM-1 |
| Mphg | CD40 |
| Mphg | CD62E/E-Selectin |
| Mphg | CD69 |
| Mphg | CXCL8/IL-8 |
| Mphg | IL-1alpha |
| Mphg | M-CSF |
| Mphg | sIL-10 |
| Mphg | SRB |
| Mphg | SRB-Mphg |
