# Supplementary material for: Development of a Topical Treatment for Psoriasis Targeting RORγ: From Bench to Skin
Source: PLoS One. 2016 Feb 12;11(2):e0147979. doi: 10.1371/journal.pone.0147979 (PMC4752338; doi:10.1371/journal.pone.0147979)
Supplement: S3 Appendix — (DOCX) [file pone.0147979.s003.docx]

**S3 Appendix.**

**
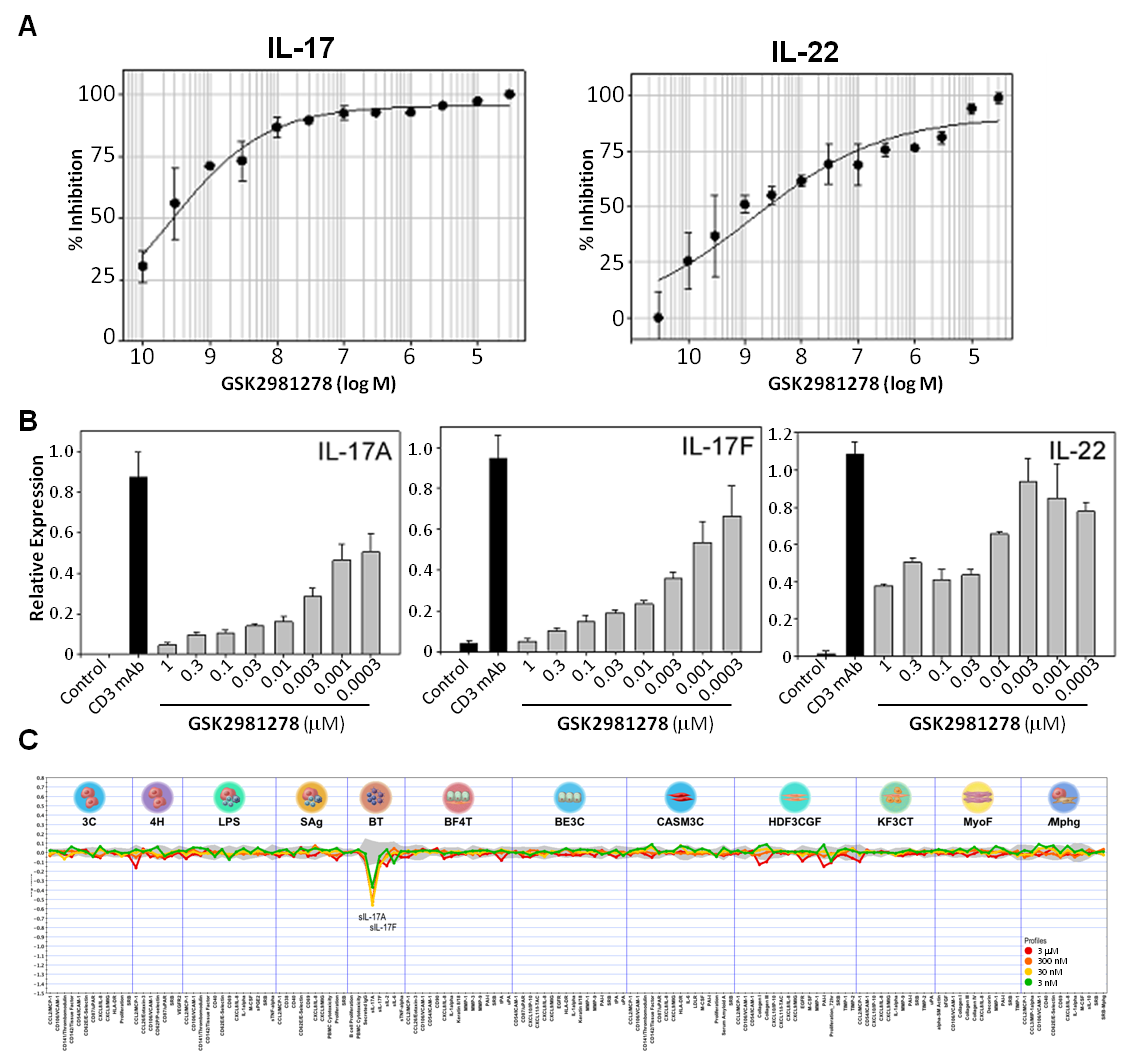
**

**GSK2981278 potently and selectively inhibits IL-17 and IL-22 levels.** CD4+ T cells were treated with compound (1 pM – 10 μM) during Th17 polarizing culture. Following the 5 day treatment period, (A) the concentration of IL-17A and IL-22 protein in conditioned medium was determined. In this study, IL-17F protein was not specifically measured because the IL-17A readout includes some cross-reactivity with the IL-17A/F heterodimer protein. (B) Transcript levels of *il17A*, *il17F* and *il22* were measured by qPCR and normalized to control TCR-stimulated cells without compound. Data shown is the percent inhibition (mean +/- SD) from a single experiment and is representative of at least 2 independent experiments. (C) GSK2981278 selectively inhibits only IL-17A and IL-17F out of 148 biomarker readouts examined in the BioMAP® Diversity Plus System™. A list of all biomarkers analyzed is presented in S2 Appendix. Data are presented as log expression ratios (log10[compound/buffer control]) of treated samples relative to solvent (DMSO) controls. Each data point represents a single well. The gray area above and below the dashed line indicates the 95% significance envelope of DMSO negative controls.
