# Supplementary material for: Development of a Topical Treatment for Psoriasis Targeting RORγ: From Bench to Skin
Source: PLoS One. 2016 Feb 12;11(2):e0147979. doi: 10.1371/journal.pone.0147979 (PMC4752338; doi:10.1371/journal.pone.0147979)
Supplement: S4 Appendix — (DOCX) [file pone.0147979.s004.docx]

**S4 Appendix.**


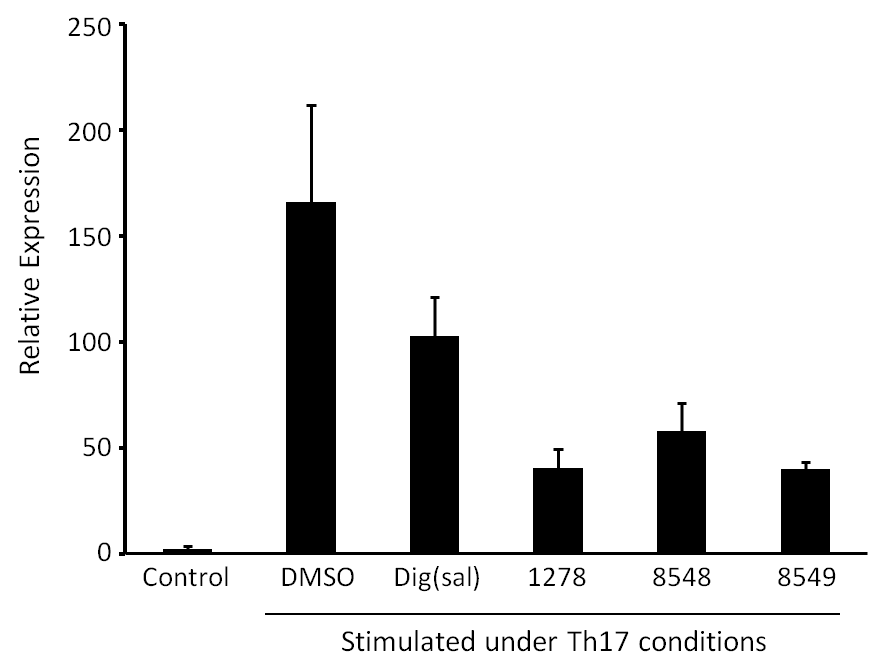


**RORγ inverse agonist reduces sRICA-induced *il17a* transcript levels in ex vivo human skin.** A pilot study using a single skin donor (three replicates per condition) evaluated whether control inhibitor, digoxin-21-salicylidene [Dig(sal)] or compounds [GSK2981278 (1278), GSK3038548 (8548) or GSK3038549 (8549)] could impact sRICA-induced il17a gene expression. Skin sections/biopsies were incubated with compounds (10 μM) for 24 hrs followed by stimulation under Th17-skewing conditions in the presence of DMSO alone or RORγ inhibition for an additional 24 hrs. Upon harvest, *il17a* RNA expression was assessed by qPCR. Graph shows the mean +/- SEM for triplicate samples from a single representative donor. GSK2981278 (1278) has been run in >20 independent studies (representing >20 distinct skin donors) with similar results.
